# Imaging findings of malignant skin tumors: radiological–pathological correlation

**DOI:** 10.1186/s13244-022-01205-8

**Published:** 2022-03-22

**Authors:** Masaya Kawaguchi, Hiroki Kato, Yoshifumi Noda, Kazuhiro Kobayashi, Tatsuhiko Miyazaki, Fuminori Hyodo, Masayuki Matsuo

**Affiliations:** 1 Department of Radiology, Gifu University, 1-1 Yanagido, Gifu, 501-1194 Japan; 2 Department of Pathology, Gifu University, Gifu, Japan; 3 Department of Radiology, Frontier Science for Imaging, Gifu University, Gifu, Japan

**Keywords:** Skin neoplasms, Magnetic resonance imaging, Positron emission tomography, Computed tomography

## Abstract

Sometimes, radiologists encounter malignant skin tumors (MSTs) during image interpretation. As MSTs require different clinical management modalities for each histological subtype, accurate preoperative diagnosis is essential. The histological subtypes of MST can be easily assessed by visual inspection or biopsy. Therefore, the significant role of radiological imaging in MSTs is to evaluate the extent of local invasion, nodal involvement, and distant metastasis, and the histological estimation of MSTs by radiological imaging has not been reported until a few years ago. However, recent studies have revealed characteristic radiological features for differential diagnosis of MSTs, such as configuration, intratumoral homogeneity, signal intensity, cyst formation, and hemorrhage. Other important clinical data for determining the histological subtype of MST include age, gender, and site of occurrence. MSTs can be categorized as epidermal, melanocytic, adnexal, and mesenchymal tumors based on the origin and have distinctive characteristics. Hence, this review article was designed to describe the clinical and radiological features of MSTs.

## Key points

- Malignant skin tumors can be categorized as epidermal, melanocytic, adnexal, and mesenchymal tumors.
- Radiological examinations play significant roles both in estimating tumor extension and in evaluating tumor characteristics.
- MRI is helpful in assessing the configuration and intratumoral characteristics, such as homogeneity, signal intensity, cyst formation, and hemorrhage.

## Introduction

Malignant skin tumor (MST), classified into melanoma and non-melanoma, is a common type of malignancy with high incidence rates worldwide [1]. The three most common histological subtypes include cutaneous basal cell carcinomas (cBCCs), cutaneous squamous cell carcinomas (cSCCs), and cutaneous malignant melanomas (cMMs). Skin is the largest organ of the body, covering approximately 16% of the total body weight, and is organized into two primary layers: the epidermis and dermis. The epidermis is the peripheral layer of the skin, which is composed of cornified, granular, spinous, and basal layers. The dermis underlies the epidermis and anchorages cutaneous structures, such as hair follicles, nerves, sebaceous glands, and sweat glands [2]. cBCCs and cSCCs originate from the epidermal layer, whereas adnexal tumors occur in the dermal layer (Table 1). Mesenchymal tumors occur in the dermis or subcutaneous tissue layer.

**Table 1:** Classification of malignant skin tumors

Layer	Origin	Malignant tumor
Epidermis	Keratinocytic/epidermal tumors	Actinic keratosis, Bowen’s disease
		Basal cell carcinoma
		Squamous cell carcinoma
		Merkel cell carcinoma
Dermis	Appendageal tumors	Proliferating trichilemmal tumor
	Follicular differentiation	Trichilemmal carcinoma
		Pilomatrical carcinoma
	Sebaceous differentiation	Sebaceous carcinoma
		Porocarcinoma
	Apocrine and eccrine differentiation	Hidradenocarcinoma
		Microcystic adnexal adenocarcinoma
		Apocrine carcinoma
	Site-specific tumors	Extramammaly Paget disease
	Melanocytic tumors	Melanocytic tumors in intermittently sun-exposed skin
		Melanocytic tumors in chronically sun-exposed skin
		Acral melanoma
	Fibroblastic tumors	Dermatofibrosarcoma protuberans
Dermis/subcutaneous tissue	Tumors of hematopoietic and lymphoid origin	Mycosis fungoides
		Sézary syndrome
	Soft tissue tumors	Atypical lipomatous tumor
	Adipocytic tumors	Pleomorphic liposarcoma
	Vascular tumors	Cutaneous angiosarcoma

In MSTs, MRI examinations are sometimes performed to evaluate tumor extension, including invasion depth. ^18^F-fluorodeoxyglucose (FDG)-positron emission tomography (PET)/CT is a useful tool for evaluating nodal and distant metastases. Previously, the prediction of histological subtypes using MRI is not required by radiologists because the MRI features of MSTs had not been reported and radiologists are unfamiliar with MSTs. Furthermore, an experienced dermatologist can easily estimate the histological subtype of MSTs by visual inspection and tissue biopsy. Although radiological investigation of MSTs has been recently published [3–6], only a few review articles have reported the MRI features of MSTs [7, 8]. Therefore, this review article was designed to describe the imaging characteristics of MSTs with emphasis on MRI features.

## Epidemiology

The prevalence and common histological subtype of MST vary according to geographical location. The number of Japanese patients with skin cancer reached 24,079 cases, accounting for 19.0 per 100,000 individuals in 2018 [9]. Alternatively, one in five Americans will develop skin cancer by the age of 70 years. Among histological subtypes, cMMs are the most common in Western countries, whereas non-melanotic skin cancers are more common than cMMs in Asia [10]. cBCCs and cSCCs comprise most non-melanotic skin cancers, accounting for 80–85% and 10%, respectively. Excluding cBCC, cSCC, and cMM, the remaining MSTs, including appendageal, hematolymphoid, and soft tissue tumors, account for only 2% of all MSTs [11].

**Fig. 1:**
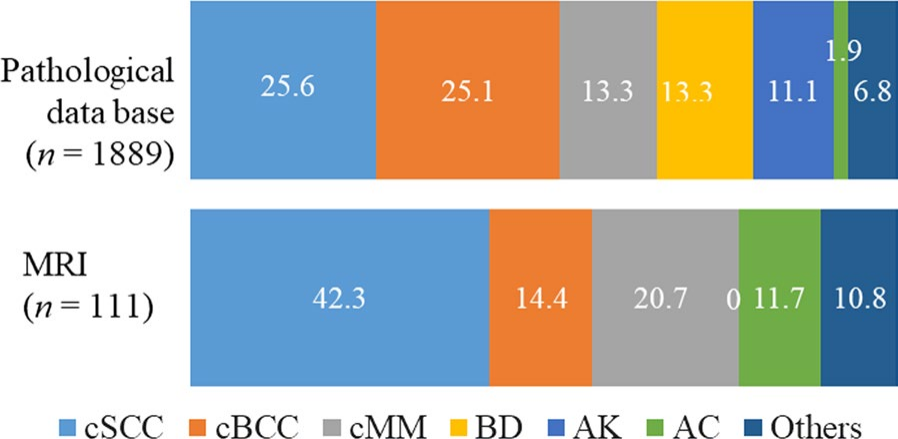
Pathological and MRI database in our institution. SCC = squamous cell carcinoma, BCC = basal cell carcinoma, MM = malignant melanoma, BD = Bowen’s disease, AK = actinic keratosis, AC = appendageal cancer

Figure 1 shows the histological and MRI database of patients with MST in our institution. The MRI databases include primary MSTs but exclude metastatic or recurrent skin tumors. In the histological database, cSCCs and cBCCs are the most common subtypes, followed by cMMs, actinic keratosis, and Bowen’s disease. Alternatively, in the MRI database, approximately half of MSTs are SCCs. No patients with actinic keratosis and Bowen’s disease underwent MRI because they are small and precursor lesions of cSCC [12].

## Anatomy

The skin is composed of three layers: the epidermis, dermis, and subcutaneous tissue [13]. The outermost layer, the epidermis, consists of a specific constellation of cells known as keratinocytes. The middle layer, the dermis, fundamentally comprises the fibrillar structural protein known as collagen. The innermost layer, subcutaneous tissue or panniculus, contains small lobes of fat cells known as lipocytes [13]. On radiological imaging, the epidermis and dermis are indistinguishable from each other because they exhibit nonspecific soft tissue density on CT and isointensy relative to the muscles on MRI [14]. Skin thickness (epidermis plus dermis) varies between the different regions of the body surface; for example, the skin of the eyelid, prepuce, and inguina is the thinnest, whereas that of the back, buttock, and sole is the thickest [15]. The subcutaneous tissue shows hypodensity on CT and hyperintensity on T1- and T2-weighted images because of its fat content [14].

## Location

The occurrence site is useful information to estimate the pathological diagnosis of MST. Sun exposure is a major risk factor in most MSTs, such as cBCC, cSCC, cMM with sun-exposed skin, and Merkel cell carcinoma (MCC), hence they often occur in sun-exposed areas (head and neck regions and upper extremity). Acral melanomas occur in the feet or hands. Proliferating trichilemmal tumors (PTTs) and cutaneous angiosarcomas (cASs) are often found in the scalp. Almost all cases of extramammary Paget disease (EMPD) are found in the skin of the genital or perianal area. Dermatofibrosarcoma protuberans (DFSP) is preferentially located on the trunk.

## Imaging

The diagnosis of MSTs using radiological examinations has limitations, because preoperative diagnosis is usually made by biopsy or visual inspection. Radiological examinations are usually performed to assess the local extent of these lesions paying particular attention to adjacent bone or deep soft tissue involvement or perineural, lymphatic, or vascular invasion is suspected, as it may alter treatment strategy. In the National Comprehensive Cancer Network guidelines, local extension of high-risk cSCC and cBCC (larger than 2 cm in the trunk or extremities and any size in the face, hands, and feet) is recommended to be evaluated by CT or MRI [16, 17]. Because of its higher sensitivity and superior soft tissue contrast, MRI is preferred over CT if perineural disease or deep soft tissue involvement is suspected [18]. MRI is a useful tool for assessing tendon, muscle, or bone marrow invasion; in contrast, CT can reveal cortical bone invasion.

### Ultrasound

Ultrasound is helpful for assessing differential diagnosis and depth of invasion, especially in small or thin lesions less than 10 mm [19, 20]. Using very-high-frequency ultrasound, ultrasound thickness of the lesion has closely correlated with pathological thickness [19]. Nevus can be distinguished from cutaneous lesions (BCCs or MMs) with high area under the curve values of over 0.9 [21].

### CT

On CT, most MSTs show non-specific soft tissue density due to its poor soft tissue contrast, and it is difficult to make an accurate diagnosis of MSTs. However, CT is sensitive in detecting calcifications, ossification, and adipose tissue. Although intratumoral fat is not observed in malignant epithelial skin tumors, it is seen in liposarcoma [22]. Only a few MSTs are accompanied by calcification or ossification. PTTs and malignant trichilemmal tumors often have calcification [23], whereas cBCCs have pathological calcification in rare cases [24]. Therefore, CT is the preferred modality to assess bone invasion, nodal metastasis, or distant metastasis.

### MRI

Table 2 shows characteristics findings of MSTs. MRI provides excellent soft tissue contrast and is helpful for assessing configuration, intratumoral homogeneity, signal intensity, cyst formation, and hemorrhage. Flat elevated lesions are observed in cSCC, cMM, and cAS [3, 25]; widespread distribution in EMPD and cAS [26]; multiple skin lesions in MCC, cAS, and cutaneous lymphoma [5]; superficial depression in cSCC and cAS [4]; and pedunculated configuration in adnexal tumors such as porocarcinoma [27, 28]. Because MSTs often show heterogeneous signal intensity on T2-weighted images, however homogeneous signal intensity on T2-weighted images is a characteristic of cBCC, porocarcinoma, and cutaneous lymphoma [3, 28]. Intratumoral hyperintensity relative to the muscle or dermis on T1-weighted images is observed in cMM and porocarcinoma [4, 28]. Intratumoral cystic foci on T2-weighted images can be seen in cBCC and porocarcinoma. A cystic lesion with a mural nodule or wall thickening has been reported in hidradenocarcinoma and cSCC arising from an epidermoid cyst [29]; and hemorrhage in cAS [25].

**Table 2:** Summary of characteristic imaging findings of malignant skin tumors

Characteristic imaging finding		Malignant skin tumor
Calcification		PTT, cBCC
Configuration	Flat elevated lesion	cSCC, cMM, cAS
	Widespread	EMPD, cAS
	Multiple skin lesions	MCC, cAS, Cutaneous lymphoma
	Pedunculated	Porocarcinoma
Intratumoral signal	Homogenous signal on T2WI	cBCC, Porocarcinoma, Cutaneous lymphoma
	Intratumoral hyperintensity on T1WI	cMM, Porocarcinoma
	Intratumoral hypointensity on T2WI	PTT, cAS
	Intratumoral cystic foci on T2WI	cBCC, Porocarcinoma
	Hemorrhage	cAS
Cystic lesion with mural nodule or wall thickening		Hidradenocarcinoma, cSCC in the epidermoid cyst

PTT = proliferating trichilemmal tumor, cBCC = cutaneous basal cell carcinoma, cutaneous squamous cell carcinoma, cMM = cutaneous malignant melanoma, cAS = cutaneous angiosarcoma, EMPD = extramammaly Paget disease, MCC = Merkel cell carcinoma

### MRI protocol

Consideration of the field of view (FOV) is important for accurate diagnosis. A FOV should be narrowed as much as possible because the lesion is solitary mass in most cases, but multiple or widespread lesions can be observed on rare cases. Flat or purpura lesions, such as cAS, are usually broader than inspection; therefore, a large FOV may be necessary. Generally, the axial plane should be heavily relied on because it enables the most specific determination of the involved tissue and association with adjacent tissues [30]. At least two planes, including axial and oblique sagittal or coronal planes, should be obtained.

The conventional MRI sequences include T1-, T2-, and fat-suppressed T2-weighted images. T1-weighted images can determine the extent of invasion into subcutaneous fat and the presence of fat or hemorrhage. T2-weighted images are usuful for evaluating the configuration and intratumoral signal intensity. Fat-suppressed T2-weighted images can detect peritumoral fat stranding and invasion of adjacent tissues. Diffusion-weighted images are limited because susceptibility artifacts occur at the air-tissue interfaces and accumulation of knowledge about apparent diffusion coefficient (ADC) of MSTs is not enough. Although the values of contrast-enhanced images are still equivocal, tumor invasion into subcutaneous fat and skull is clearly visible on contrast-enhanced images in cAS on the scalp [31].

Recent studies establish the advantage of high-resolution (HR) microscopy-coil MRI [32–34]. HR-MRI considerably improves the spatial resolution and signal-to-noise ratio. The depth of tumors measured by HR-MRI has a strong correlation with histopathological depth, and HR-MRI provides an accurate prediction of the involvement of the bone, cartilage, subcutaneous fat, or muscle [33, 34]. Although the utility of HR-MRI for diagnosing MSTs has not yet been established, HR-MRI is expected to enhance microstructural characterization such as superficial depression, tumor margins, and microcystic foci.

### ^18^F-FDG-PET/CT

^18^F-FDG-PET/CT provides both morphologic information and data on tumor metabolic activity. ^18^F-FDG-PET/CT is also a useful tool for detecting nodal and distant metastases as in other malignancies; it can unmask subtle recurrences or micrometastases [7]. A systematic review of cMMs has reported that the sensitivity of PET and PET/CT ranged from 68 to 87% and their specificity ranged from 92 to 98% in patients with stage III and IV diseases [35].

## Malignant skin tumors

### Cutaneous basal cell carcinoma (cBCC)

cBCC, which arises from keratinocytes near the basal layer, is the most common malignancy of skin cancer, and its incidence is rising [36]. cBCC derives from the basal cell layer and outer root sheath of hair follicles [37]. Most cBCCs occur on skin areas exposed to ultraviolet radiation from sunlight, with 75%–85% of lesions found in the head and neck regions. cBCCs most commonly occur during the seventh decade of life and affect men more frequently than women [3]. Nodal or distant metastases are extremely rare (0.0016–0.1343%) [38].

Histological subtypes of cBCCs are categorized as follows: low risk (e.g., nodular, superficial, and pigmented cBCC) and high risk (morpheaform and infiltrative cBCC). Nodular BCC is the most common subtype of cBCCs and characterized by nests of basaloid cells with sharp borders. The presence of bulky mucin aggregates can produce a cystic structure [37].

**Fig. 2:**
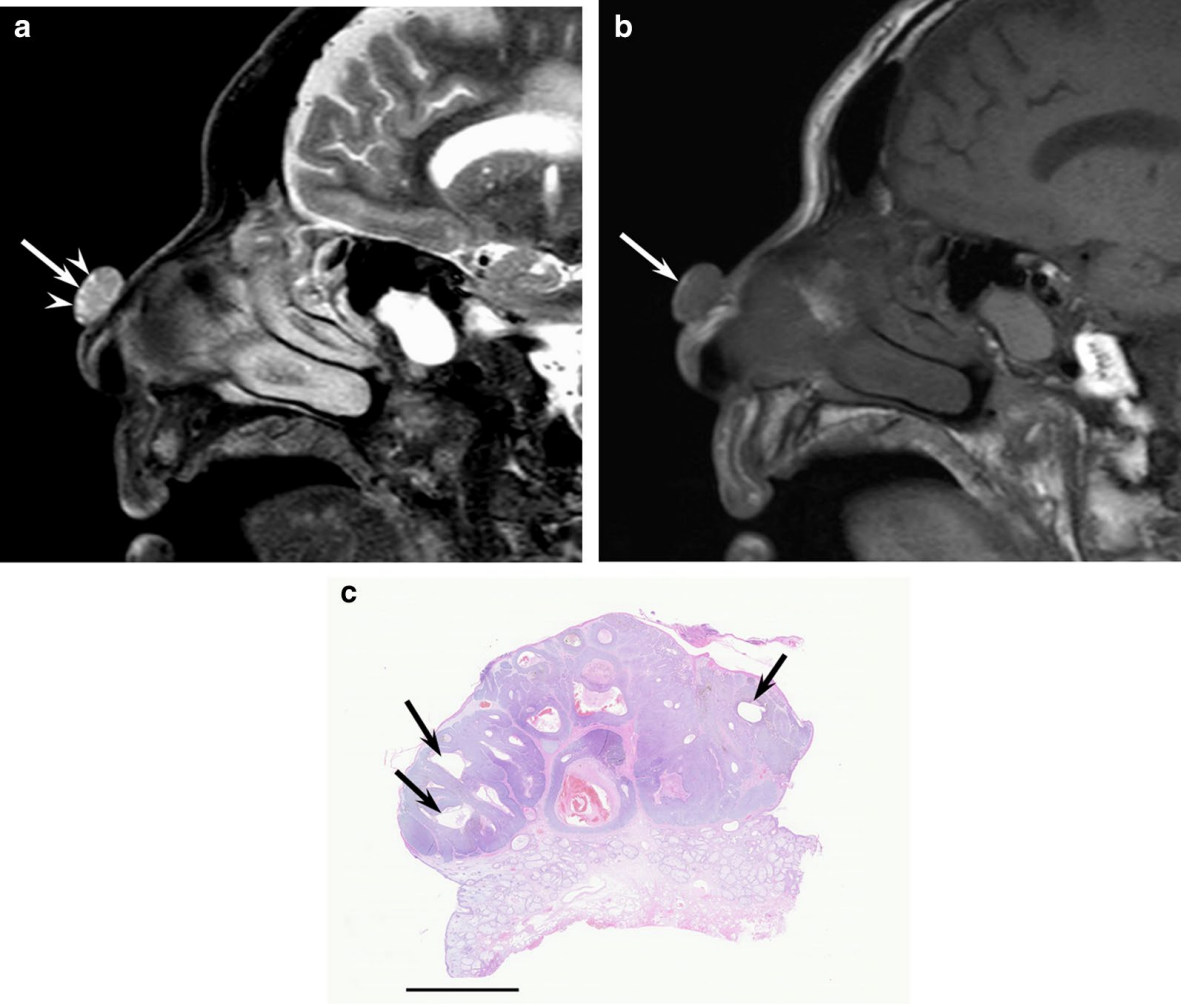
An 86-year-old male with basal cell carcinoma on the nose. **a** Sagittal fat-suppressed T2-weighted image showing an elevated oval mass (arrow) with cystic foci (arrowheads). **b** Sagittal T1-weighted image showing an oval mass (arrow). **c** Histological specimen (H&E stain; scale bar, 5 mm) showing a well-defined mass in the dermis with multiple cystic cavities with mucinous contents (arrows)

MRI findings of nodular cBCCs in the head and neck regions show elliptical cutaneous lesions occurring mainly in the nose. These lesions have well-demarcated deep tumor margin without peritumoral fat stranding or protruding into subcutaneous tissue, with a mean maximum diameter of 23.5 mm [3]. On T2-weighted images, they show homogeneous signal intensity accompanied by intratumoral T2-hyperintense foci (Fig. 2). Homogeneous signal intensity reflects homogeneous growth pattern without necrosis, whereas T2-hyperintense foci correspond to cystic cavities filled with mucinous contents [3]. Routine ^18^F-FDG-PET/CT imaging for assessing nodal or distant metastasis is not recommended as the probability of metastasis is low in cBCCs [39].

### Cutaneous squamous cell carcinoma (cSCC)

cSCC, which is derived from epidermal keratinocytes, is the second most common non-melanoma skin cancer after cBCC [3]. The most significant risk factors for cSCC include sun exposure, age, and immunosuppression. cSCC affects mainly sun-exposed areas, such as the face, scalp, neck, arms, and hands [40]. The incidence of cSCC increases with age, with an average age of onset of the mid-60 s. cSCC is more common in men than women (3:1 ratio) [41]. The estimated incidence rates of lymph node metastasis and disease-specific death are 3.7–5.2% and 1.5–2.1%, respectively. Tumor diameter of more than 2.0 cm is the risk factor most highly associated with disease-specific death [41].

The histological investigation of cSCC reveals strands of atypical keratinocytes originating from the epidermis and infiltrating into the dermis or subcutaneous tissue [42]. cSCCs range from well-differentiated SCCs, which show minimal pleomorphism and prominent keratinization, to poorly differentiated cSCCs, which show a high degree of atypia, frequent mitoses, and few keratin horn pearls [42].

**Fig. 3:**
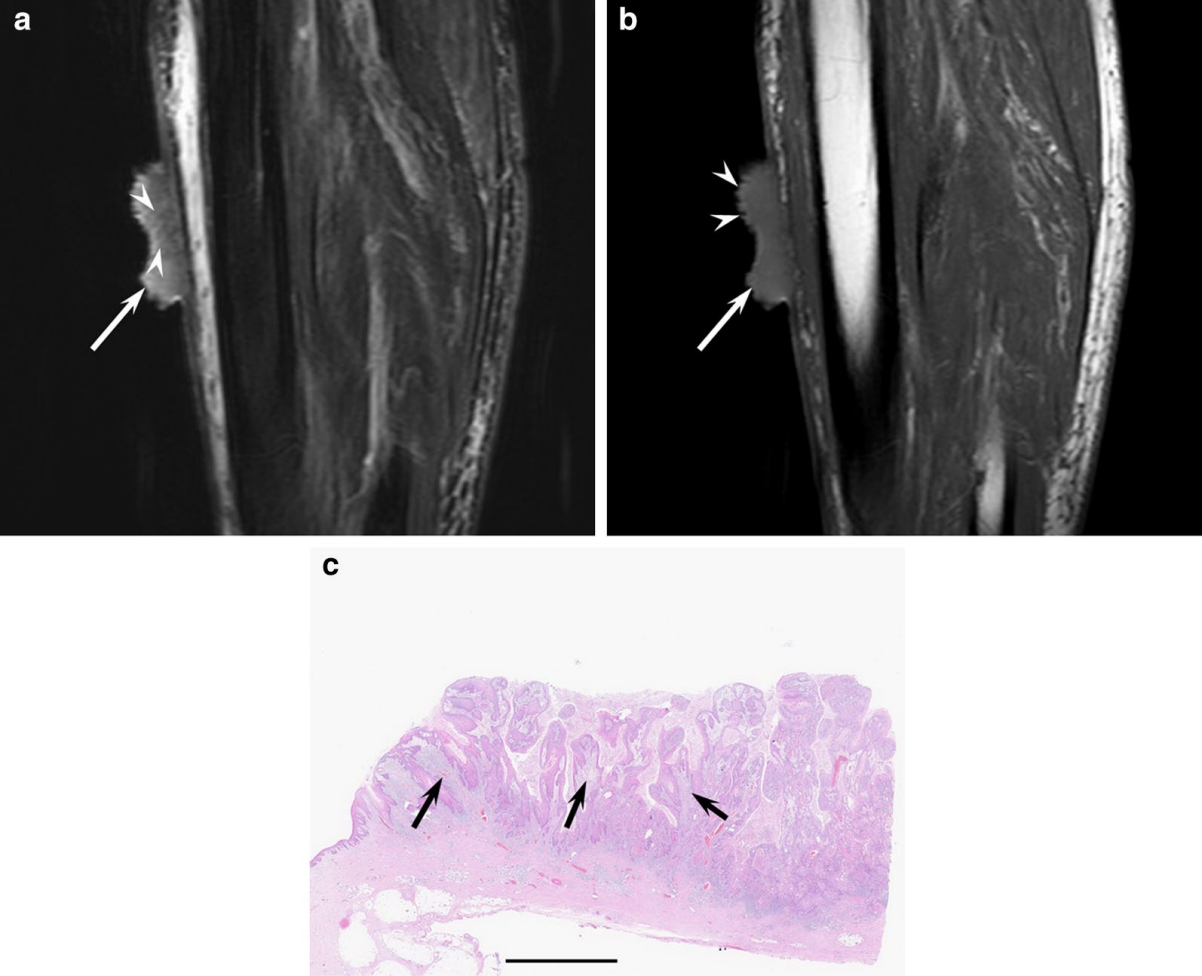
An 86-year-old male with squamous cell carcinoma on the lower leg. **a** Sagittal fat-suppressed T2-weighted image showing an elevated and flattened mass (arrow) with superficial ulcer formation and linear hyperintensity (arrowheads). **b** Sagittal T1-weighted image showing a mass (arrow) with superficial irregular margins (arrowheads). **c** Histological specimen (H&E stain; scale bar, 5 mm) showing invasive squamous cell carcinoma nests surrounded by stromal components with infiltration cells (arrows)

On MRI, cSCCs show flattened lesions with a maximum diameter of 23.5–39.5 mm [3, 4]. Superficial ulcer formation, protrusion into subcutaneous tissue, ill-demarcated deep tumor margin, and peritumoral fat stranding are often observed, reflecting more aggressive behavior in cSCCs than in cBCCs [3]. Reticular or linear hyperintensity and heterogeneous signal intensity on T2-weighted images and superficial irregular margins are observed due to a mixture of tumor cells and surrounding stroma with inflammatory cells and loose fibrosis (Fig. 3). ^18^F-FDG-PET/CT is recommended as clinically indicated to rule out distant metastatic disease [18]. ^18^F-FDG-PET/CT has high sensitivity for detecting primary and recurrent cSCC lesions, including small cutaneous and nodal disease, which can lead to changes in management in 22–28% of all cases [6, 43]. The maximum standardized uptake value (SUVmax) of the primary cSCC is higher than in cSCC with recurrence than in cSCC without recurrence (13.0 vs. 6.4) [44].

### Cutaneous malignant melanoma (cMM)

cMM is a malignant tumor arising from melanocytes and primarily involves the skin. cMMs can affect the eye, meninges, and various mucosal surfaces [45]. The incidence of cMM varies greatly between countries, due to variations in racial skin phenotype. Most cMMs in northern hemisphere populations occur in sun-exposed skin areas; however, some types of cMMs occur in skin areas without sun exposure, and these tumors account for most cMMs in non-White populations [46]. cMMs arising from sun-exposed skin areas usually occur in the head and neck, upper extremities, and trunk. Alternatively, acral melanomas, which are representative cMMs arising from sun-shielded sites, occur in the subungual area, sole of the foot, and palm of the hand [45, 47]. Although cMMs are associated with poor prognosis, the 5-year overall survival is reaching 52% in patients with cMM using nivolumab-plus-ipilimumab [48].

Histopathologically, the pathway of cMMs is mainly classified into two phases: radial and vertical growth phase. Radial growth phase is defined as an atypical melanocytic proliferation confined to the epidermis and superficial dermis. Vertical growth phase is a melanocytic proliferation in the dermis characterized by expansile growth (tumorigenic proliferation) and/or mitotic activity [46]. While cMMs are usually heavily pigmented, they can also be amelanotic [45].

**Fig. 4:**
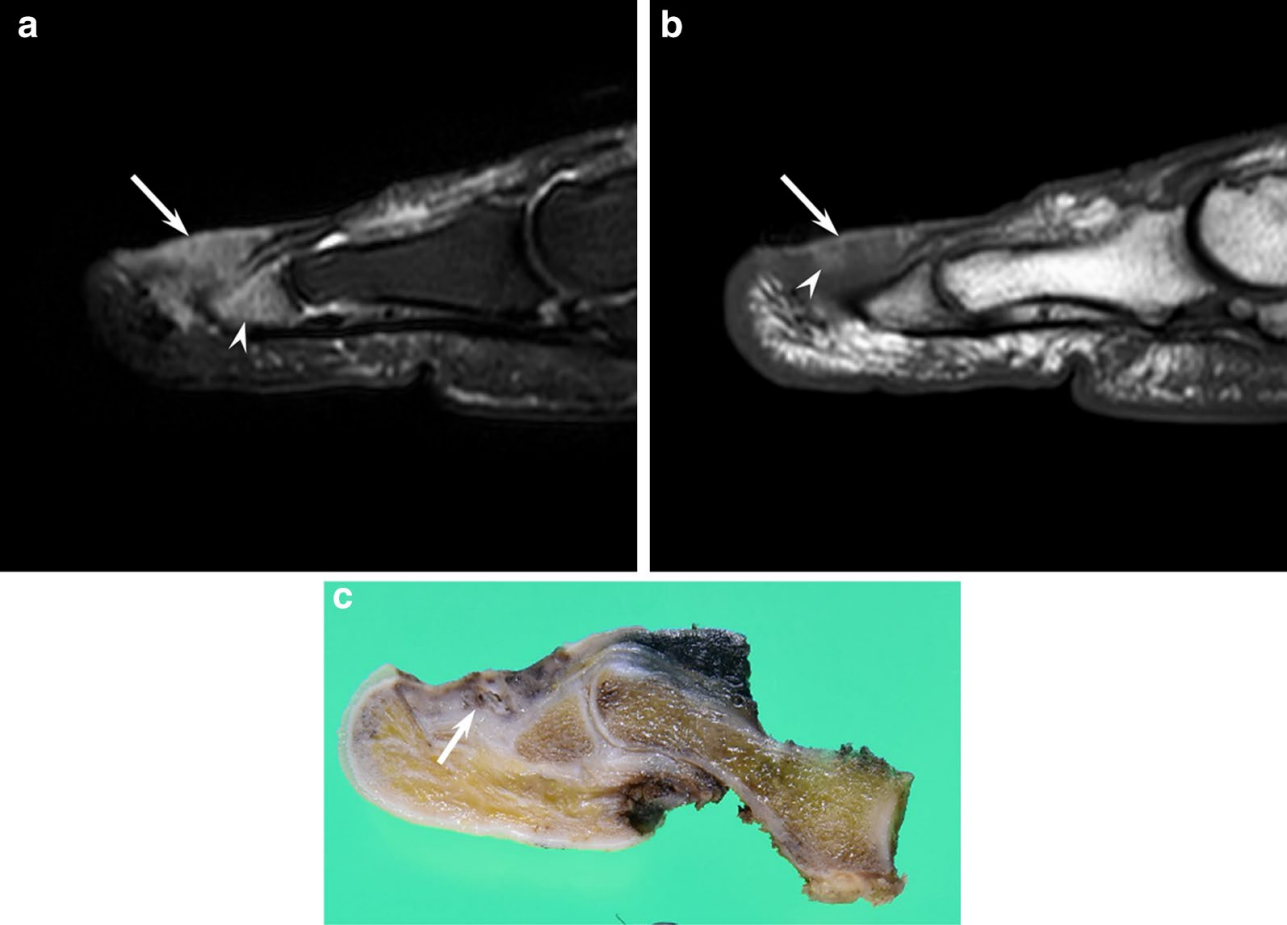
A 74-year-old male with malignant melanoma on the toe. **a** Sagittal fat-suppressed T2-weighted image showing a flattened subcutaneous mass (arrow) with bone marrow edema-like signal (arrowhead). **b** Sagittal T1-weighted image showing an isointense mass (arrow) with intratumoral hyperintensity. **c** Macroscopic specimen (cut surface) showing intratumoral hemorrhage or melanin deposition (arrow)

cMMs appear as flat elevated lesions with ill-defined deep tumor margins and peritumoral fat stranding, reflecting subcutaneous fat tissue invasion [4]. Although superficial depression is rarely seen on MRI, it indicates poor prognosis due to being consistent with pathological ulceration [4, 46]. On T1-weighted images, approximately half of cMMs show hyperintensity relative to the dermis due to melanin deposition or intratumoral hemorrhage (Fig. 4). In contrast, the remaining half of cMMs shows isointensity relative to the dermis [4]. On ^18^F-FDG-PET/CT, a higher SUVmax of ≥ 2.2 of primary lesions is significantly associated with recurrence [49, 50].

### Porocarcinoma

Porocarcinoma is a rare type of skin cancer arising from eccrine sweat glands; it is composed of poroid and cuticular cells [51, 52]. The risk factors for developing porocarcinoma are still not well understood. The most common affected site is the head and neck, followed by the lower extremity [51]. The mean age is 67.5 years without gender predilection. Masses and nodules are the most common modes of presentation [51]. Metastases occur at presentation in 31% of all cases; the most common metastasized sites are the nearby lymph nodes (58%), followed by the lung (13%) [51, 52].

Histopathologically, porocarcinoma evolves from a preexisting poroma, which is the benign counterpart of porocarcinoma; however, it can also develop de novo [51]. Several histological features include ductal differentiation, nests of basaloid cells, nuclear pleomorphism, and atypical mitoses [51, 52].

**Fig. 5:**
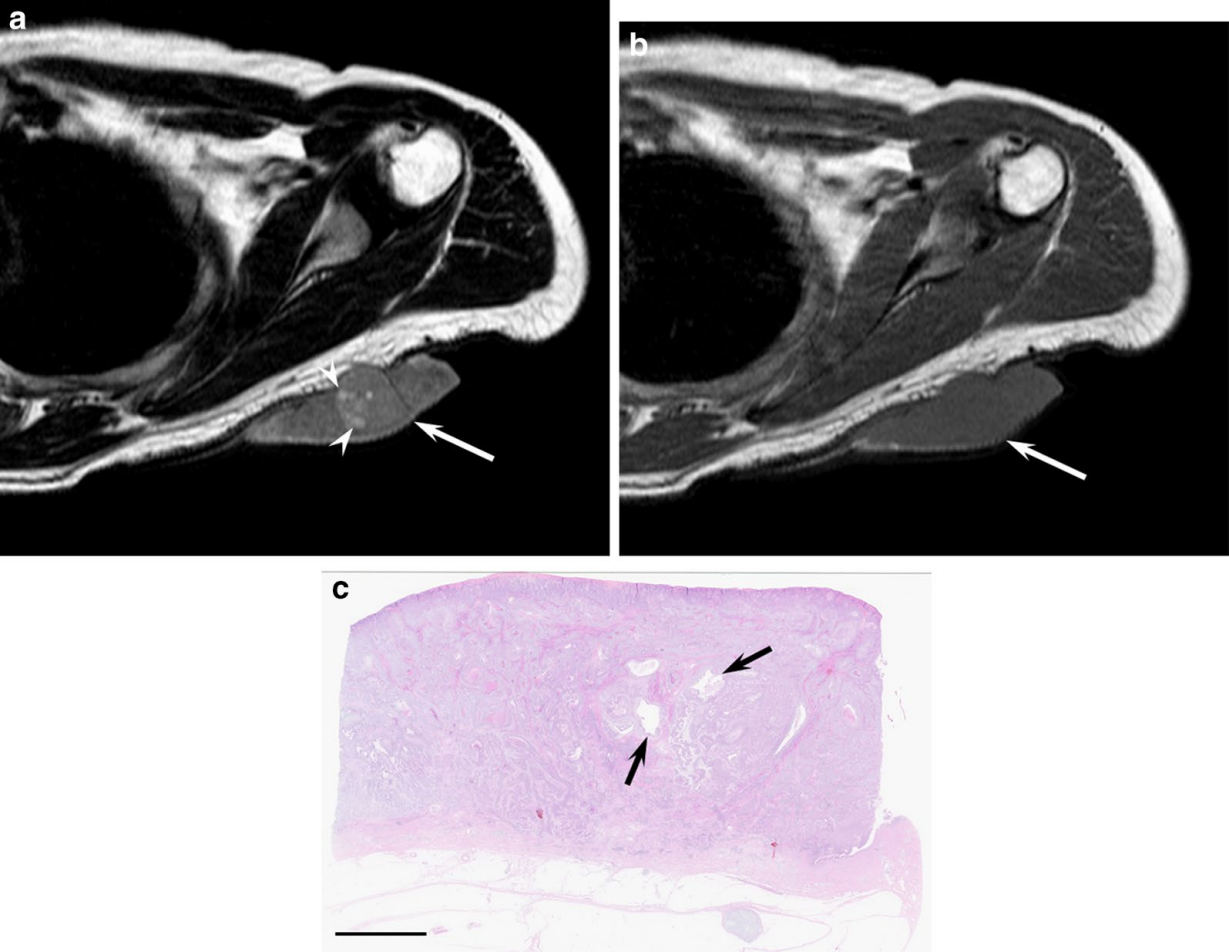
A 60-year-old male with porocarcinoma on the upper back. **a** Axial T2-weighted image showing a pedunculated mass (arrow) with T2-hyperintese foci (arrowheads). **b** Axial T1-weighted image showing slight hyperintensity compared with muscle. **c** Histological specimen (H&E stain; scale bar, 5 mm) showing a well-defined mass in the dermis with multiple cystic areas (arrows)

On MRI, porocarcinomas usually appear as pedunculated solid masses; meanwhile, in rare instances, porocarcinomas appear as subcutaneous cystic masses. The mean maximum diameter and height of solid masses are 36 mm and 12 mm, respectively [28]. Moreover, well-demarcated tumor margins and smooth skin surfaces are observed. On T2-weighted images, solid components demonstrate mild hyperintensity relative to the muscle, accompanied by hyperintense foci filed with fluid contents due to ductal differentiation (Fig. 5). On T1-weighted images, intratumoral hyperintensity relative to the muscle is observed, reflecting richly vascular stoma with dilated vessels [28].

### Hidradenocarcinoma

Hidradenocarcinoma is a rare malignant adnexal tumor arising from the sweat glands, constituting the malignant counterpart of hidradenomas [53, 54]. Hidradenocarcinomas occur most frequently on the head and neck region, especially on the face [53]. Lesions are solitary, varying in size ranging from 1 to 6 cm [54]. There is a slight male predominance; it occurs most often in the fifth to the seventh decades of life. Nodal and visceral metastases occur in 39% and 28% of all cases, respectively. The recurrence rate is as high as 50%, and the 5-year postsurgical survival rate is less than 30% [53].

Tumors arise conventionally de novo, or the carcinoma might occur due to the overgrowth of the benign precursor lesion. Hidradenocarcinomas are composed of the same cell types as in hidradenomas, including predominantly clear cells, squamoid cells, and mucinous cells [54]. Malignant cells show nuclear pleomorphism, necrosis, and increased mitotic activity [54].

**Fig. 6:**
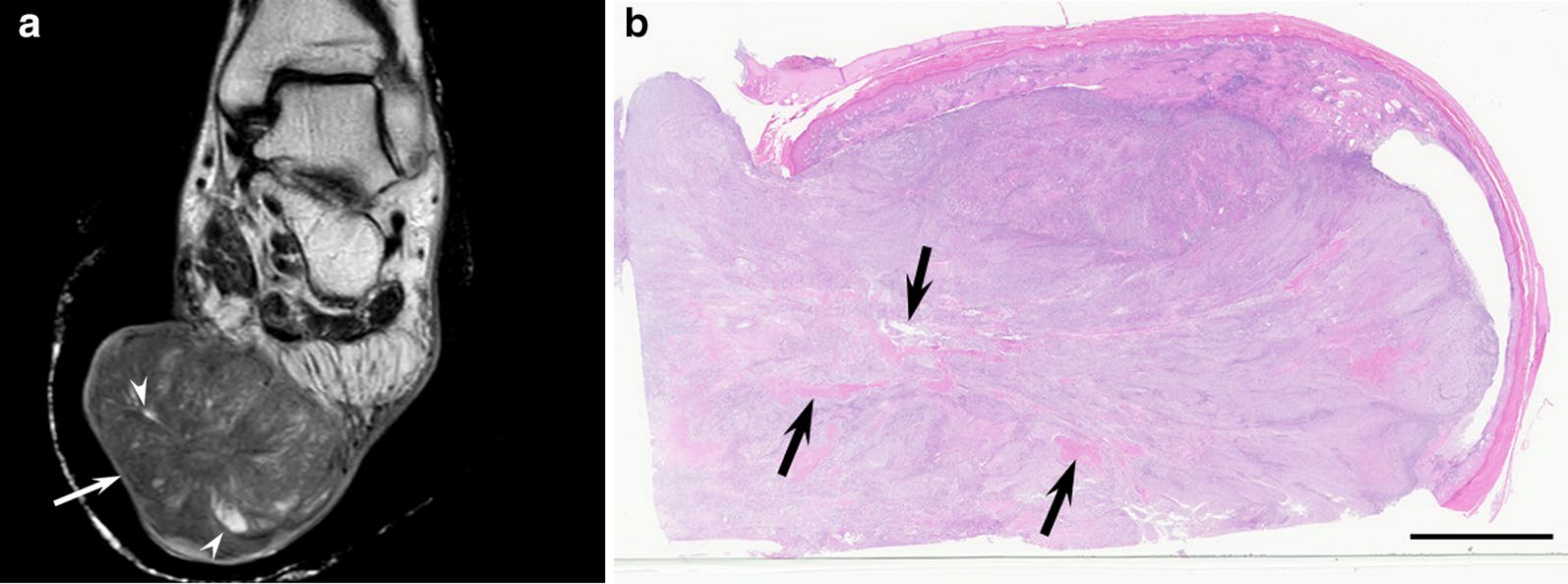
A 66-year-old male with hidradenocarcinoma on the sole. **a** Sagittal T2-weighted image showing an elevated huge mass (arrow) with different-sized hyperintense foci (arrowheads). **b** Histological specimen (H&E stain; scale bar, 5 mm) showing a mass with multiple necrotic areas (arrows)

The characteristic imaging features of hidradenocarcinomas are not yet determined. Based on previous case reports, hidradenocarcinomas are radiologically classified into two patterns: cystic lesions with mural nodules or solid lesions with cystic components [29, 55]. The former shows a well-circumscribed oval mass attached to the dermis. Hyperintensity or hypointensity of internal fluid contents relative to the muscle on T1-weighted images is caused by hemorrhage or necrotic mucoid fluid. Mural nodules show prominent enhancement on contrast-enhanced T1-weighted images [29, 55]. The latter appears as a subcutaneous solid mass with heterogeneous signal intensity, reflecting cysts or necrosis [56] (Fig. 6). The size of the cysts varies from small to large.

### Extramammaly Paget disease (EMPD)

EMPD is a rare cutaneous malignancy, presenting as a primary intraepithelial adenocarcinoma. EMPD commonly occurs on apocrine-rich skin including Paget cells and usually affects the skin of the genital or perianal area [57, 58]. EMPD usually occurs between the ages of 50 and 80 years, with a peak incidence at the age of 65 years, with a male predilection in Asian populations [26, 58]. EMPD often presents with a slowly enlarging, asymmetrical, white and red, scaly plaque. Patients with EMPD have a good prognosis with a 5-year overall survival rate of 75–95% [59].

Histopathologically, EMPD is characterized by intradermal proliferation of Paget cells. Typical findings include large round Paget cells singly or in nests, distributed throughout the epidermis [57]. Paget cells are concentrated in the lower epidermis but can scatter higher. They mainly spread horizontally; however, in advanced stages, invasion is deeper. Moreover, invasion into the dermis, subcutaneous tissue, blood vessels, and lymphatic vessels is observed [26, 58].

**Fig. 7:**
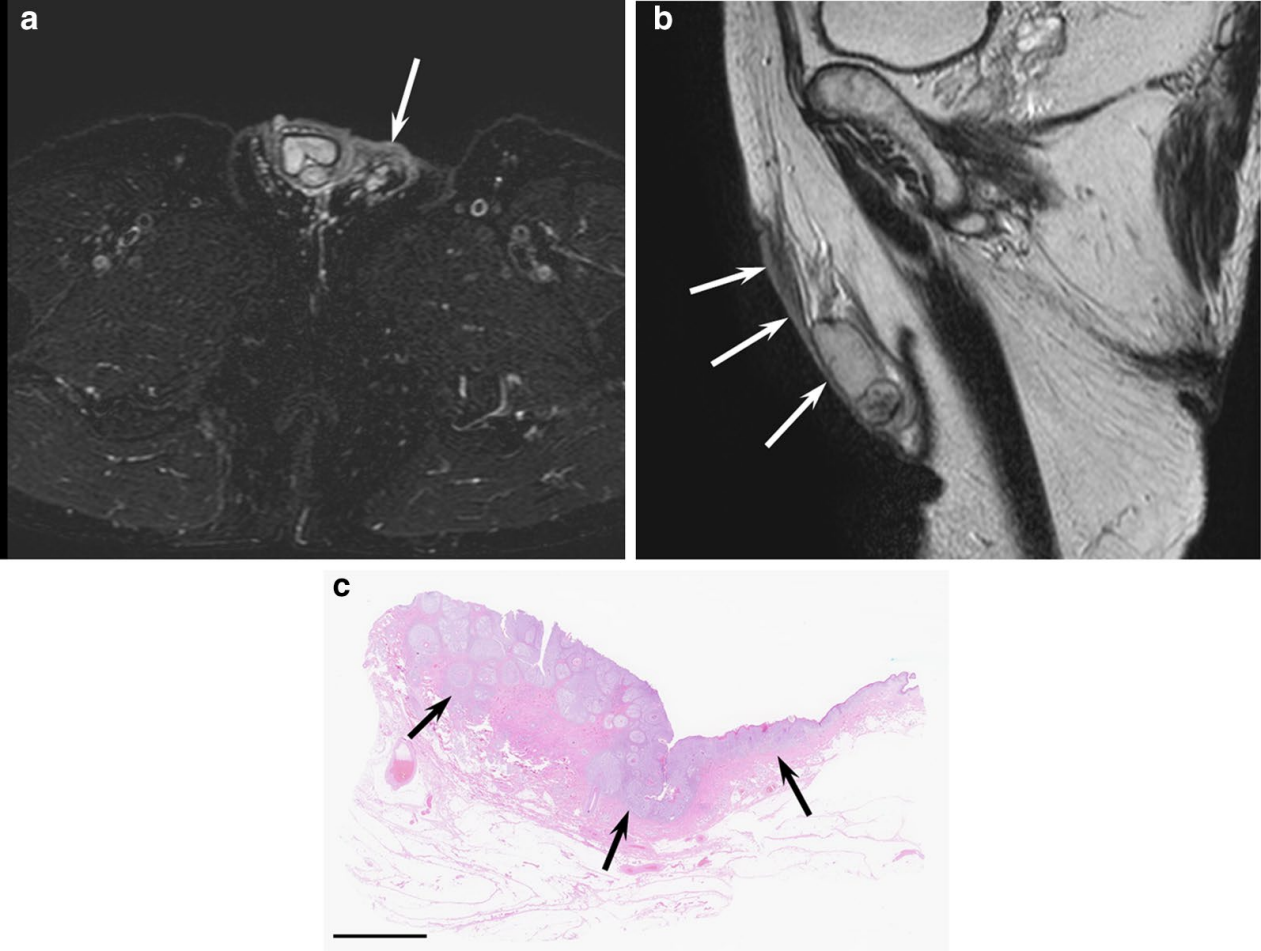
A 77-year-old male with extramammary Paget disease on the perineal region. **a** Axial T2-weighted image showing a hyperintense cutaneous mass (arrow). **b** Sagittal T2-weighted image showing a cutaneous mass (arrows) with horizontal spread. **c** Histological specimen (H&E stain; scale bar, 5 mm) showing a horizontally spreading mass within the dermis (arrows)

On MRI, EMPDs are demonstrated as horizontally widespread skin thickness due to spreading Paget cells along the epidermis and dermis. T1- and T2-weighted images show nonspecific hypointensity (Fig. 7). Marked enhancement on gadolinium-enhanced T1-weighted images [26]. A good correlation in vertical and horizontal ranges of tumor extension between histological examination and gadolinium-enhanced T1-weighted images has been reported [26]. On ^18^F-FDG-PET/CT, thick lesions (> 1.0 cm) show intense FDG uptake (SUVmax, 7.5–14.9), whereas thin lesions (< 1.0 cm) show mild FDG uptake (SUVmax, 3.0–4.0) [60].

### Proliferating trichilemmal tumor (PTT)

PTT is a solid-cystic neoplasm with predominant outer root sheath differentiation at the isthmus [61]. PTT originates from the external root of hair follicles and occurs preferentially in areas with dense hair follicle concentration; therefore, PTTs usually affect the scalp, accounting for 85%. Patient ages range from 21 to 88 years, with a mean age of 62.4 years [62]. The ratio of males to females is 1:4. PTT usually appears as a solitary, slow-growing, and exophytic lesion with a size of 2–25 cm. Some lesions show rapid growth of a previously existing asymptomatic lesion [61]. The morphological spectrum includes benign, atypical (intermediate), and rare malignant lesions [61]. The rates of local recurrence and regional lymph node metastasis are 3.7% and 1.2%, respectively [62].

PTT occurs either de novo or from an existing trichilemmal cyst, the benign counterpart of PTT. PTT appears as a well-circumscribed solid and cystic neoplasms involving the dermis and sometimes extending to the subcutaneous tissue. Intratumoral calcification, ample eosinophilic cytoplasm, and keratinization without a granular layer (trichilemmal keratinization) are often observed [61].

**Fig. 8:**
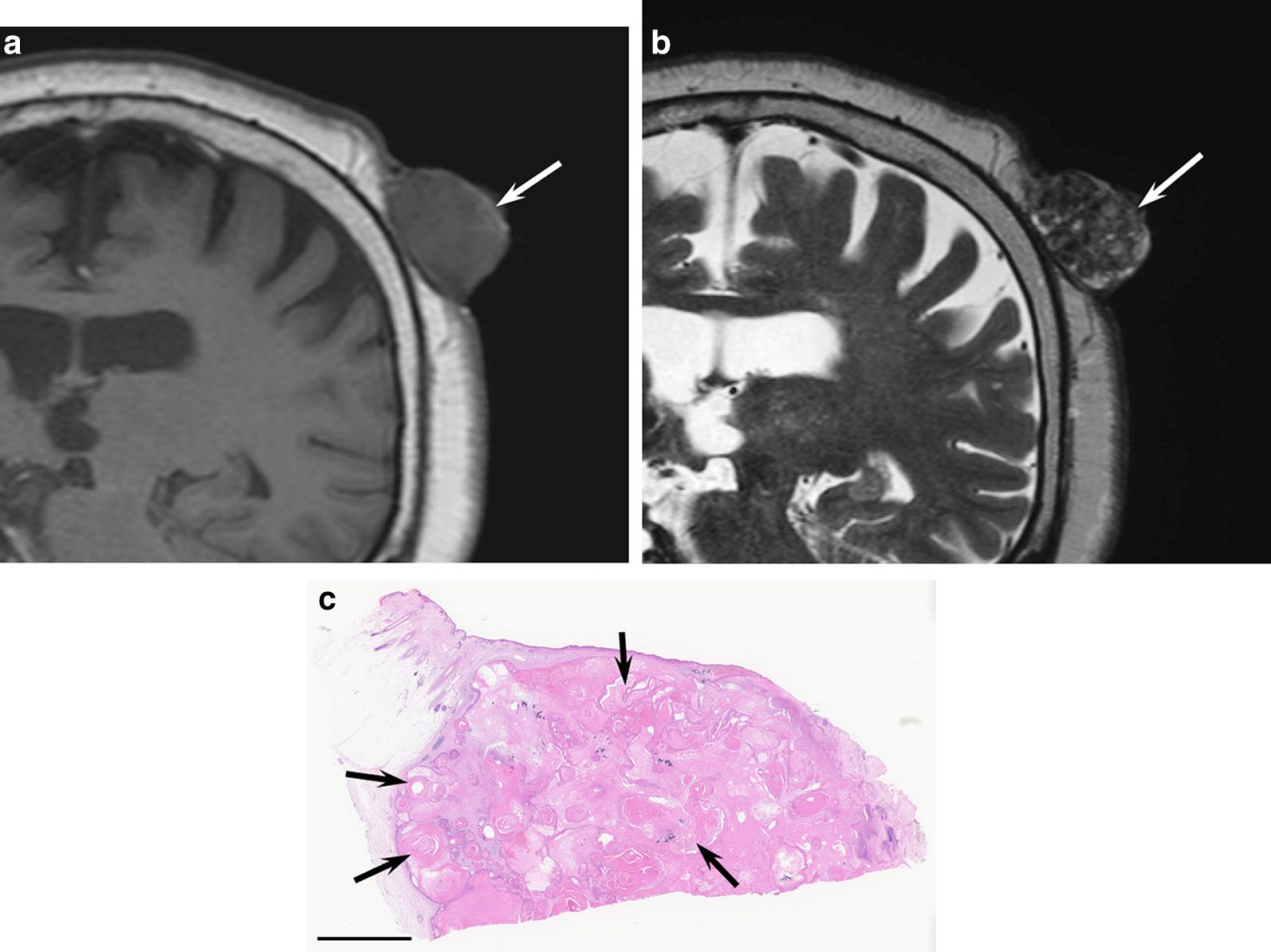
A 81-year-old female with a proliferating trichilemmal tumor on the scalp. **a** Oblique sagittal T1-weighted image showing an elevated mass with nonspecific signal intensity (arrow). **b** Oblique sagittal T2-weighted image showing heterogeneous hypointensity (arrow). **c** Histological specimen (H&E stain; scale bar, 5 mm) showing a well-circumscribed mass with multiple keratinization (arrows)

The configuration of PTT is typically oval/round shape. On CT, PTTs show soft tissue density masses and are often associated with intratumoral calcification and hyperdense areas, which reflect a characteristic histological finding of trichilemmal keratinization with dystrophic calcification and abundant cholesterol crystals [23]. On T2-weighted images, they show heterogeneous signal intensity with intratumoral hypointense areas relative to the gray matter, which corresponds to a layered and aggregated keratin debris [23] (Fig. 8). Marked restricted diffusion is not usually observed with a relatively high ADC value of 1.49 ± 0.24 × 10^−3^ mm^2^/s) [23].

### Sebaceous carcinoma (SC)

SC is a rare and potentially aggressive cutaneous malignancy arising from sebaceous glands [63]. Areas with particularly high-density sebaceous glands (i.e., eyelids, face, scalp, and neck) have a higher incidence of SC. SCs most commonly occur in the periorbital region [64]. SCs primarily affect elderly individuals, with a median age of 72 years. Men and women are affected equally. The most common presentation of periorbital SC is a painless subcutaneous nodule of the eyelid. The rate of regional nodal involvement in ocular SC may be as high as 10%–28%. Distant metastasis is uncommon; however, cases with metastasis to the lung, liver, bone, and brain have been reported [63, 64].

Histopathologically, sebaceous glands with variable degrees of differentiation and atypia are observed. Growth patterns may be lobular, focal necrosis, papillary, or mixed. SC demonstrates a basaloid neoplasm in lobules or sheets of cells separated by a fibrovascular stroma with infiltrating edges [63]. Lipid granules are present in the cytoplasm of cells and are positive for oil red O stain.

**Fig. 9:**
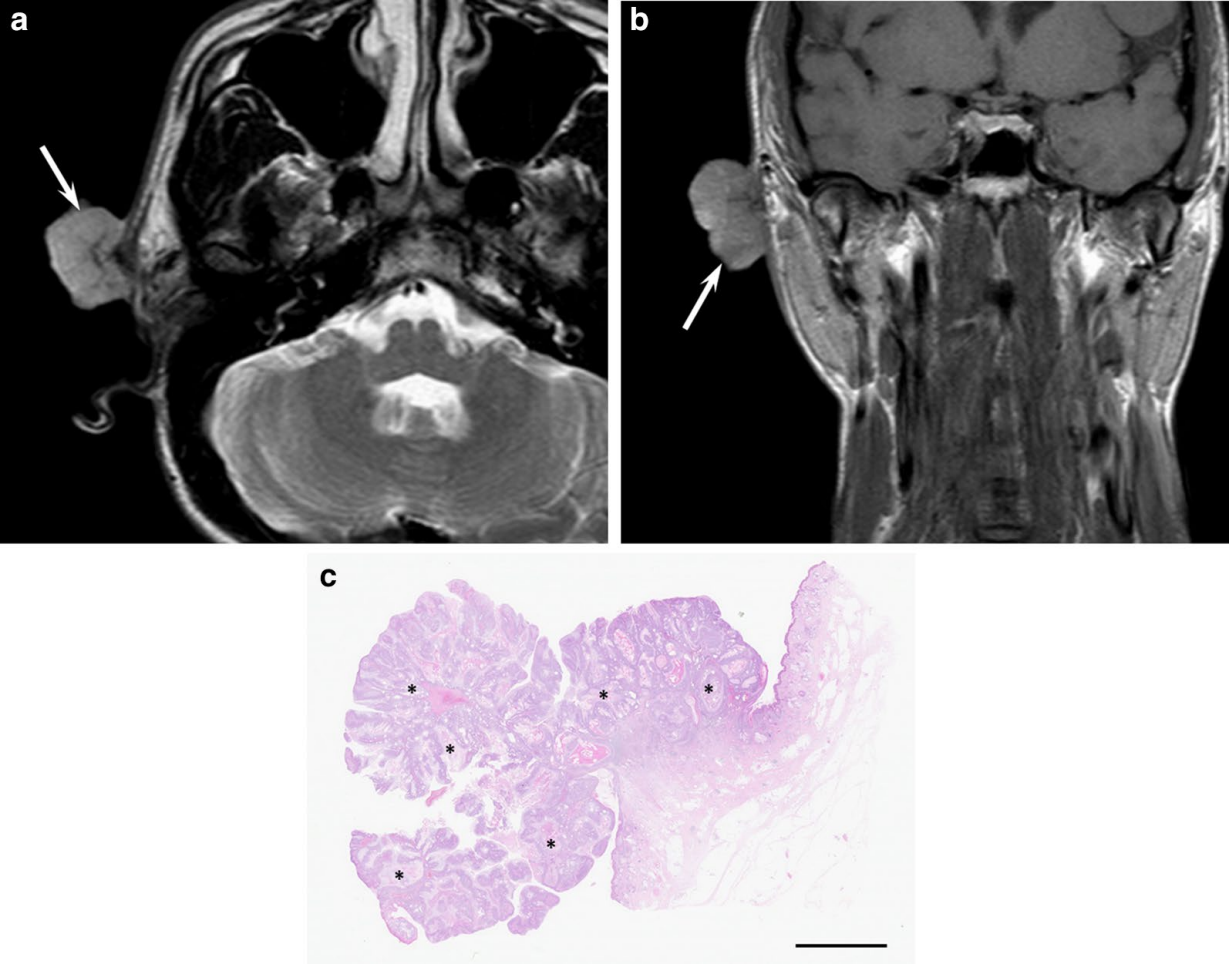
A 38-year-old female with sebaceous carcinoma on the cheek. **a** Axial T2-weighted image showing a pedunculated mass with hyperintensity (arrow). **b** Coronal T1-weighted image showing a hyperintense mass relative to the muscle (arrow). **c** Histological specimen (H&E stain; scale bar, 5 mm) showing a pedunculated mass with interventional sebocytes (asterisks)

Detailed imaging findings of SCs have not been reported. SCs show either a pedunculated or invasive mass. In the eyelid, they show small elevated nodules in the early stage and invade into the orbit as the disease progresses [65, 66]. Slight hypodensity relative to the muscle on CT and hyperintensity on both T1- and T2-weighted images may be seen because the lesion contains lipid materials (Fig. 9).

### Merkel cell carcinoma (MCC)

MCC is an aggressive neoplasm defined as a primary neuroendocrine carcinoma of the skin [67]. Most MCCs are driven by Merkel cell polyomavirus, and the reminding MCCs are associated with exposure to ultraviolet light [68]. Most tumors are located in the head and neck (48%), followed by the upper extremity (25%) [69]. MCC tends to affect men more than women at a rate of 2:1, commonly occurring in individuals older than 50 years [9]. The typical clinical presentation of MCCs is an erythematous or violaceous nodule on sun-exposed areas [9]. The most clinically relevant prognostic factors include tumor size and the presence of regional or distant metastasis [68]. The 5-year overall survival is approximately 51% for local disease, 35% for nodal disease, and 14% for distant disease [9].

Histopathologically, MCCs are typically characterized as dermal tumors with sheets and nests of small round cells. Numerous mitotic figures and necrotic cells are commonly observed [9]. Because of high cellularity, small round cell tumors, such as blastic lymphomas, should be considered in the differential diagnosis [67].

**Fig. 10:**
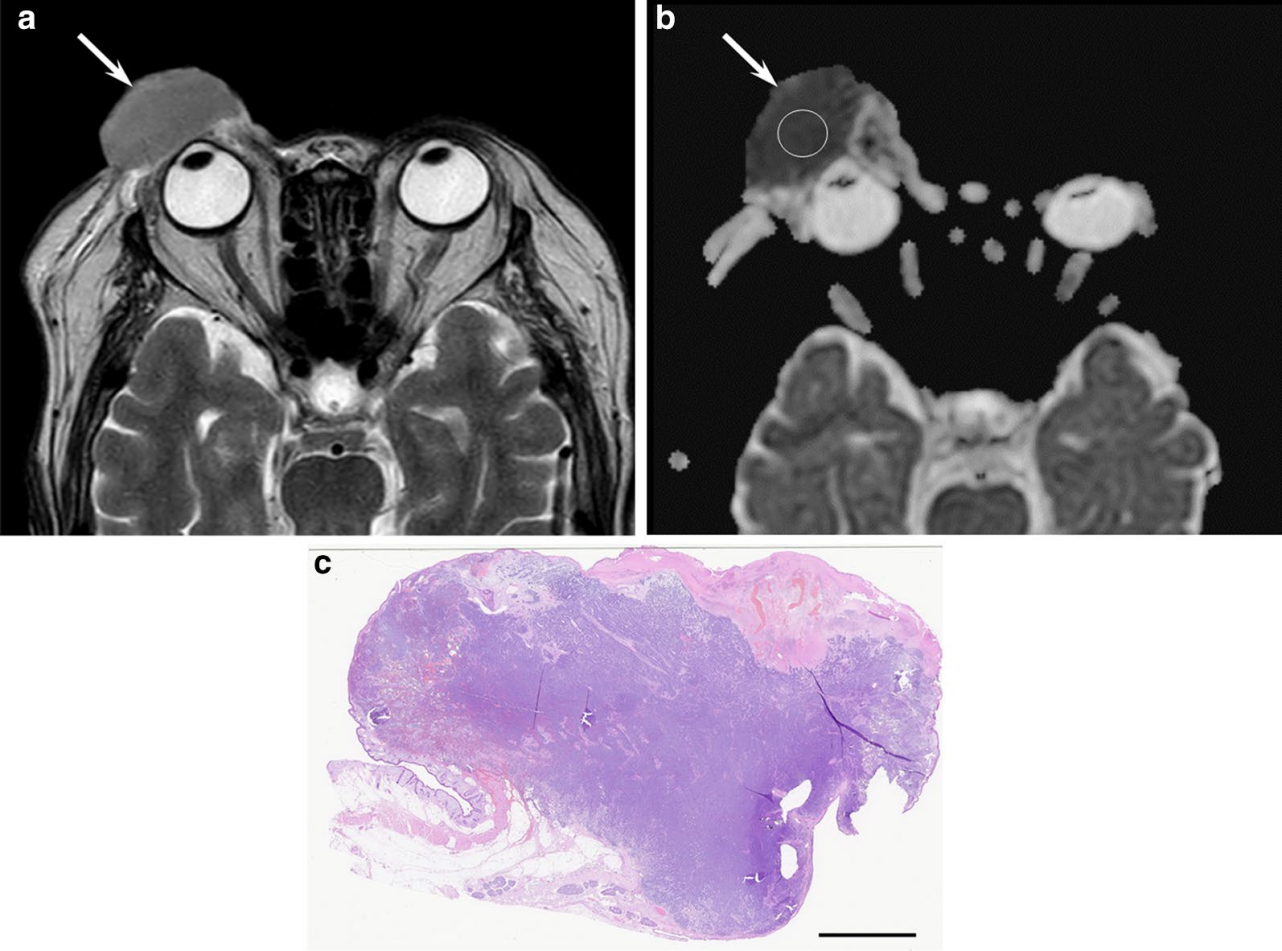
A 79-year-old female with Merkel cell carcinoma on the eyelid. **a** Axial T2-weighted image showing an elevated cutaneous mass with homogeneously mild hyperintensity (arrow). **b** Axial apparent diffusion map image showing marked restricted diffusion (0.5 × 10^−3^ mm^2^/s) (arrow). **c** Histological specimen (H&E stain; scale bar, 5 mm) showing a hypercellular mass in the dermis and subcutaneous tissue

Common imaging features of primary MCCs include cutaneous or subcutaneous nodules and focal or diffuse skin thickening [5]. Moreover, MCCs appear as perifascial muscular or intramuscular metastases [18]. Necrosis is common, whereas calcifications are unseen. On MRI, MCCs exhibit hypo- to isointensity on T1-weighted images and iso- to hyperintensity on T2-weighted images reflecting the high cellularity of small round cell tumors [5] (Fig. 10). Differential diagnosis includes cutaneous or soft tissue lymphoma owing to the increased cellularity. They show a low ADC value due to high cellular density. The mean SUVmax of ^18^F-FDG-PET for primary MCCs ranges from 4 to 6.5 [5].

### Cutaneous angiosarcoma (cAS)

cAS are a family of aggressive malignancies demonstrating vascular endothelial cell differentiation [70]. Their etiology is still unknown in most cases, although few cASs arise after radiation exposure or long-standing lymphedema as a part of Stewart–Treves syndrome [39]. The head and neck region is the most common location of cASs, with the scalp alone accounting for more than 60% of cases [25]. The estimated male-to-female ratio is approximately 3:1, with its incidence peaking in the seventh decade of life. cASs on the scalp typically present with an enlarging bruise-like purpura and may be associated with ulceration and/or mass formation [71]. The prognosis for cAS is poor, with a 5-year survival rate of less than 40%. Prognostic factors associated with poor survival are older age, worse performance status, larger tumor size, and the scalp as the site of the primary lesion [71].

Macroscopically, cASs are typically hemorrhagic with areas of focal necrosis or cystic degeneration. cASs range from well-formed, anastomosing vessels to solid sheets of high-grade epithelioid or spindle cells and high-grade morphology, with variable nuclear atypia, mitotic activity, and coagulative necrosis [39].

**Fig. 11:**
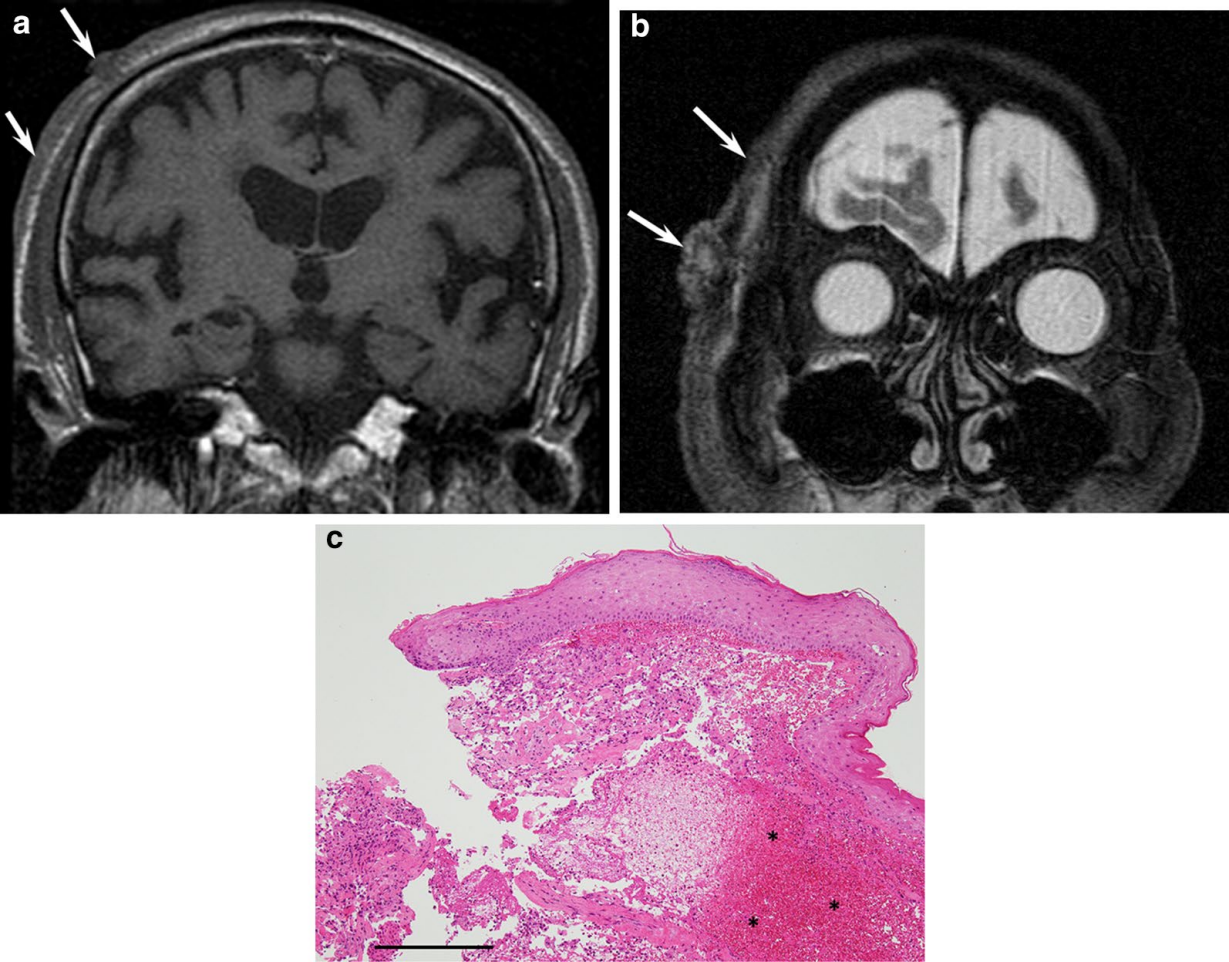
A 91-year-old male with angiosarcoma on the scalp. **a** Coronal T1-weighted image showing a horizontally spreading lesion (arrow) with a nodule (arrowhead). **b** Coronal fat-suppressed T2-weighted image showing elevated lesions with an obtuse angle with mixed hyper- and hypointensity (arrows). **c** Histological specimen (H&E stain; scale bar, 1 mm) showing atypical endothelial cells with massive hemorrhage in the dermis

On images, cASs on the scalp show the wide-ranging appearance of single or multiple lesions [25, 31]. MRI shows flattened configuration, reflecting horizontal extension, with a maximum diameter and diameter-to-height ratio of 34.9 mm and 3.3, respectively. They usually appear as flattened elevated lesions with an obtuse angle, invading the subcutaneous tissue and muscles. Intratumoral hypointensity and mixed hyper- and hypointensity on T2-weighted images are characteristic features of cASs due to intratumoral hemorrhage and hemosiderin deposition [25] (Fig. 11). On ^18^F-FDG-PET/CT, primary tumors with high SUVmax values (> 7.96) show significantly poorer prognosis than those with low SUVmax values [72].

### Dermatofibrosarcoma protuberans (DFSP)

DFSP is a relatively usual, locally aggressive cutaneous mesenchymal tumor, characterized by a high rate of local recurrence, but minimal risk of metastasis. DFSP is preferentially located on the trunk, accounting for 40–50%, followed by the proximal portion of the limbs, accounting for 30–40% [73]. It is most commonly found between the second and fifth decades of life, with a male-to female ratio of approximately 3:2. The overall survival rate of DFSP is high, ranging between 91 and 100% [73, 74].

Histopathologically, DFSP shows a poorly invading the full thickness of the dermis and extending into the subcutaneous tissue. The infiltration of tumor cells into the subcutaneous fat has been demonstrated as a honeycomb pattern. Microscopically, DFSP is characterized by a uniform population of spindle-shaped fibroblasts with long nuclei, intercellular collagen, dilated vascular space, and small capillaries [74, 75].

**Fig. 12:**
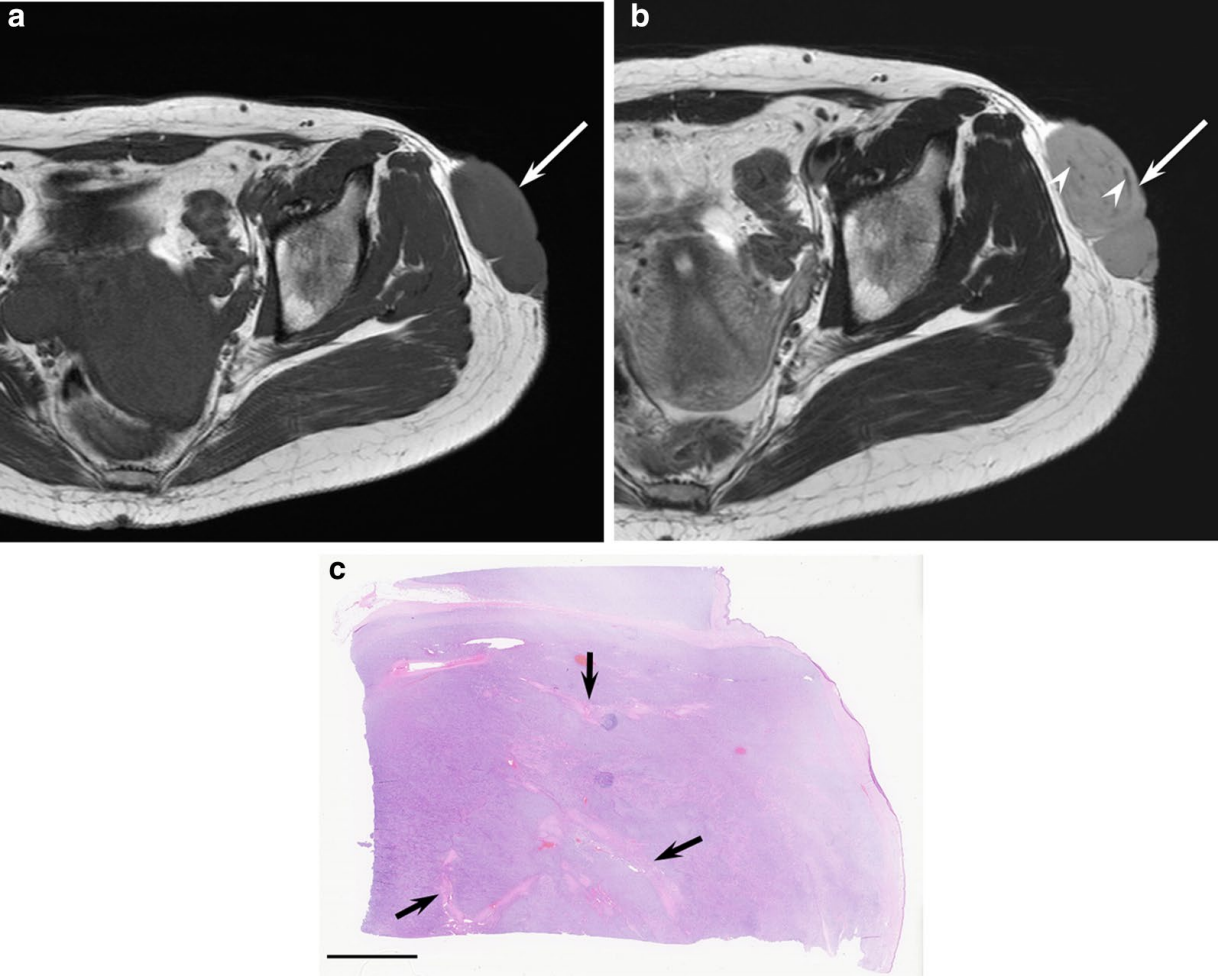
A 27-year-old female with dermatofibrosarcoma protuberans on the hip. **a** Axial T1-weighted image showing a subcutaneous mass (arrow). **b** Axial T2-weighted image showing a hyperintense mass (arrow) with linear hypointensity (arrowheads). **c** Histological specimen (H&E stain; scale bar, 5 mm) showing tumor nests with fibrous tissue in the dermis (arrows)

On images, DFSPs appear as oval and well-defined masses, but are rarely ill-defined because of the permeative infiltration into subcutaneous fat [74]. The diameter of the lesions ranges from 2 to 17 cm (mean 5.6 cm) [76]. DFSPs appear as isointense superficial nodules or masses relative to the muscle on T1-weighted images and show predominant hyperintensity on T2-weighted images owing to the presence of myxoid degeneration. However, they also show intermediate to low signal intensity on T2-weighted images due to various amounts of fibrous tissue (Fig. 12). On contrast-enhanced T1-weighted images, intermediate or marked degree of homogeneous or heterogeneous enhancement is observed due to the hypervascularity [74, 76].

### Mycosis fungoides (MF)

MF is the most common subtype of cutaneous T-cell lymphoma (CTCL), accounting for 60% of all CTCLs, and comprises almost half of all primary cutaneous lymphomas [77]. Associations with long-term exposure to various allergens and associations with chronic skin disorders have also been suggested as possible etiological factors. Common sites include the upper thigh and groin, breasts, armpits, and the crook of the elbow. Most patients are adults/elderly; however, the disease can also occur in children and adolescents, with a 2:1 male-to-female ratio [78]. In the early stage, the lesions are often confined to sun-protected areas and show patches or erythematous plaques. Patients with advanced-stage MF characteristically present with a combination of patches, plaques, and tumors, which often show ulceration [78]. Although the 5-year survival rate of patients with early disease (stage IA–IIA) is 78–94%, that of patients with advanced disease (stage IIIB–IVB) is less than or equal to 40% [79].

Histopathologically, early patch lesions show superficial band-like or lichenoid infiltrates, mainly consisting of lymphocytes and histiocytes. With progression to the tumor stage, the tumor cells increase in number and size, showing variable proportions of small, medium, and large cerebriform cells. The infiltration of these cells becomes more diffuse in the dermis and subcutaneous tissue [78].

**Fig. 13:**
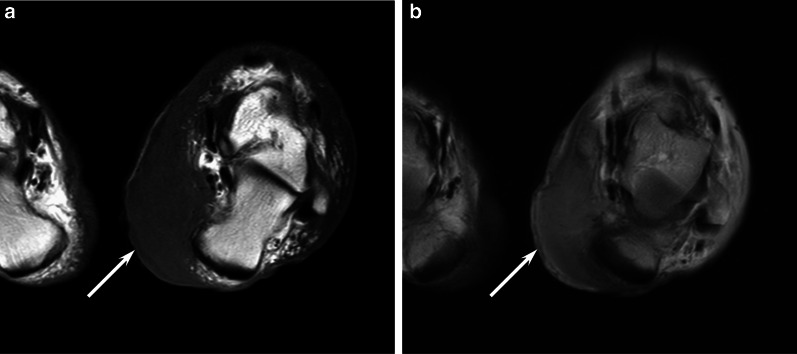
A 73-year-old female with mycosis fungoides on the foot. **a** Axial T1-weighted image showing an extensive skin thickening with a bulky subcutaneous mass (arrow). **b** Axial T2-weighted image showing a subcutaneous mass with homogeneous intermediate signal intensity (arrow)

On images, in the early stage, MFs show no abnormality or slight degree of cutaneous thickening. In the stage with tumor formation, diffuse thickening or mass formation in the skin is observed, sometimes associated with an ulcer. On T2-weighted images, MFs show homogeneous intermediate signal intensity, reflecting their high cellularity (Fig. 13). Diffuse marked enhancement on contrast-enhanced images and marked restricted diffusion on diffusion-weighted images are observed [80, 81].

## Conclusion

Radiological investigation of MSTs is essential for evaluating tumor extension, nodal involvement, and distant metastasis. Although considering the differential diagnosis of MSTs is still challenging for radiologists, we believe that MRI can play a supplementary role for determining the histological subtypes of MSTs.

## Data Availability

Data sharing is not applicable. Because this article has no datasets that were constituted or analyzed during the current study.

## Abbreviations

ADC: Apparent diffusion coefficient
cAS: Cutaneous angiosarcoma
cBCC: Cutaneous basal cell carcinoma
cMM: Cutaneous malignant melanoma
cSCC: Cutaneous squamous cell carcinoma
CT: Computed tomography
DFSP: Dermatofibrosarcoma protuberans
EMPD: Extramammary Paget disease
FDG: Fluorodeoxyglucose
HR: High-resolution
MCC: Merkel cell carcinoma
MF: Mycosis fungoides
MRI: Magnetic resonance imaging
MST: Malignant skin tumor
PET: Positron emission tomography
PTT: Proliferating trichilemmal tumor
SC: Sebaceous carcinoma
SUVmax: Maximum standardized uptake value
